# Prognostic Value of KRAS Gene Mutation on Survival of Patients with Peritoneal Metastases of Colorectal Adenocarcinoma

**DOI:** 10.1155/2021/3946875

**Published:** 2021-09-13

**Authors:** Manuel Díez-Alonso, Fernando Mendoza-Moreno, Remedios Gómez-Sanz, Belén Matías-García, Enrique Ovejero-Merino, Raquel Molina, Sonia Soto-Schütte, Alberto San Juan, Alberto Gutierrez-Calvo

**Affiliations:** ^1^Department of General and Digestive Surgery, Príncipe de Asturias Teaching Hospital, Alcalá de Henares, Madrid, Spain; ^2^Department of Oncology, Príncipe de Asturias Teaching Hospital, Alcalá de Henares, Madrid, Spain

## Abstract

**Objective:**

The main objective of the study was to determine the effect of the presence of mutation in the KRAS gene on the survival in patients with colorectal cancer (CRC) and peritoneal metastases (PM).

**Materials and Methods:**

A retrospective cohort study was performed. Patients diagnosed with CRC with synchronous or metachronous PM between January 2006 and December 2019 were included. Data on the histopathological, clinical, and treatment factors were collected. The effect of each variable on survival was evaluated by Cox regression.

**Results:**

A total of 149 patients were included (64 women (43%) and 85 men (57%); mean age, 63 years). The long-term survival rate at 36 months was 24% (median, 21 months). KRAS mutation was detected in 75 patients (50.3%). Kaplan–Meier analysis estimated that likelihood of survival was higher in patients with wild-type KRAS tumours (35%) than in mutated-type KRAS (14%) (median: 28 vs. 15, respectively) (*P*=0.001). Within the categories into which the peritoneal cancer index (PCI) was classified, survival at 36 months depended on the KRAS status. Survival in wild-type KRAS tumours with PCI 1–10 was 71% and with PCI 11–20 was 26%, while in mutant-type KRAS tumours, survival was 41% and 4%, respectively (*P*=0.025). In the multiple regression analysis, the KRAS mutation was revealed to have an independent prognostic value (HR: 2.144; 95% CI: 1.342–3.424).

**Conclusion:**

The mutational status of the KRAS gene has demonstrated a strong association with survival and prognostic utility in patients with CRC with PM.

## 1. Introduction

Peritoneal metastases (PM) are diagnosed in 10% of patients with colorectal cancer (CRC) [1]. The diagnosis can be made during follow-up, after resection of the primary tumour (metachronous metastases) (4.2% of patients) or at the same time as the primary tumour (synchronous metastases) (4.8% of patients) [2]. The peritoneum is the sole focus of metastases in 2–5% of CRCs [3], although PM is present in 30% of cases of disseminated colon cancer and in 5% of patients with disseminated rectal cancer [1, 2].

Traditionally, the presence of PM in a patient with CRC has been considered a terminal, incurable situation, susceptible only to symptomatic treatment or palliative chemotherapy. A decade ago, the median survival that could be expected in these patients was 12 months when systemic chemotherapy was administered and 6 months without it [4]. In recent years, there has been a substantial improvement in the prognosis of patients with metastatic CRC following the introduction of modern chemotherapy programs based on 5-fluorouracil + cisplatin/irinotecan [5, 6]. But, the survival of patients with PM remains lower than that of patients with metastatic spread, without peritoneal involvement [7].

In recent years, cytoreductive surgery combined with hyperthermic intraperitoneal chemotherapy (CRS/HIPEC) has become widespread for the treatment of patients with PM. A 5-year survival of more than 40% has been achieved [8]. However, this is a complex procedure, with high morbidity (16–64%) and mortality (8%), and appropriate patient selection for PM remains unclear.

The identification of predictive factors, which are associated with response to chemotherapy, and prognostic factors, which are associated with OS, is essential for selecting and planning the treatment of cancer patients. This has been extensively studied in patients with CRC metastatic to the liver or lung. However, surprisingly few studies have investigated prognostic factors in patients with PM [9, 10].

Mutations of the KRAS oncogene have been found in 30–40% of patients with colorectal liver metastases and have been associated with recurrence and poor overall survival [11]. They are now recognized as a valuable prognostic factor in this type of patients. We hypothesized that KRAS mutations, as a direct measurement of tumour biology, may be a powerful predictor of outcome also in patients with CRC with PM treated with perioperative modern chemotherapy.

## 2. Materials and Methods

### 2.1. Retrospective Cohort Study

Patients diagnosed with CRC with PM between January 2006 and December 2019 were included. The patients were selected from the data collected in the computerized file of the Coloproctology Unit, which was filled in prospectively during these years. The study was approved by the Ethics Committee of the Príncipe de Asturias Teaching Hospital. The main objective of the study was to determine the effect of the presence of a mutation in the KRAS gene on the survival of CRC patients with PM.

### 2.2. Inclusion and Exclusion Criteria

Inclusion criteria were as follows: age between 18 and 75 years, primary tumour with histopathology of adenocarcinoma, and presence of PM detected during surgery or by radiological techniques (MRI or CT Scan with HIPEC protocol (administration of intravenous contrast during intestinal phase and co-administration of 20 ml water-soluble oral contrast (Gastrografin®) diluted in 200 ml of water). Both cases of synchronous metastases, coincident with the primary tumour, and cases of metachronous metastases, detected during follow-up, were included. Exclusion criteria were as follows: ECOG (Eastern Cooperative Oncology Group) functional status greater than 2, patients deceased due to postoperative complications, age over 80 years, and synchronous tumour of another organ.

The diagnosis of PM was made by histopathological examination of biopsies obtained during surgery. In cases that did not undergo surgery, the diagnosis was based on CT and MRI findings. The extent and degree of peritoneal disease was assessed, and the PCI (peritoneal cancer index) was calculated for each patient [12]. Once the diagnosis was made, all patients were evaluated by a multidisciplinary committee.

Levels of carcinoembryonic antigen (CEA) and CA 19-9 and biochemical parameters were determined at the time of diagnosis. Thoracic, abdominal, and pelvic CT scans were performed for the staging of distant metastases. Rectal tumours were staged after MRI and endorectal ultrasound. KRAS mutational status (codons 12 and 13) was assessed using the biopsies of tumour samples, when the presence of metastases was detected, synchronically with the primary tumour or during follow-up, and the results were categorized into two groups: wild-type KRAS (WT-KRAS) and mutant-type KRAS (MT-KRAS). KRAS status was assessed when the diagnosis of metastases was made.

### 2.3. Treatment

Surgical resection of the primary tumour was indicated, as a first measure, in case of tumours producing symptomatology (obstruction, perforation, or haemorrhage). At the same time, metastasis to other organs was assessed. After the operation, chemotherapy was administered with programs based on cisplatin/irinotecan/5-fluorouracil (FOLFOX/FOLFIRI). If the patient's functional status was good and there were no major comorbidities, bevacizumab or antibodies against epidermal growth factor (anti-EGFR) (cetuximab or panitumumab) were added, depending on the KRAS mutation status. Six cycles were scheduled, and tumour response was assessed by CT and/or MRI at the end of the cycle. Peritoneal disease response was quantified. In case of PCI less than 10 and absence of metastases in other organs, CRS/HIPEC surgery was indicated. In case of nonresponse to chemotherapy, new lines of chemotherapy were scheduled or palliative symptomatic treatment was oriented according to the patient's functional status.

In the case of patients with primary tumours that did not produce symptomatology and with known and present peritoneal disease, chemotherapy was scheduled as in the previous group. At its completion, response and degree of distant disease were assessed. If the tumour response was favourable, with no metastases in other organs and the patient's functional status permitted, CRS/HIPEC was scheduled. Otherwise, chemotherapy or palliative symptomatic measures were continued, depending on each patient. In these cases, surgery was a symptomatic measure and was reserved for cases that developed complications.

### 2.4. Statistical Analysis

The results of variables related to demographic data, analytical data, location, extent of metastatic disease, histopathology, treatment, and evolution were collected. For this study, PCI was categorized into three classes: PCI 1–10, 11–20, and > 20. Variables were collected in a spreadsheet of Microsoft Excel 2019 (v.19). Statistical analysis was performed by SPSS program (v.23) (IBM, Armonk, New York, USA). The present study first described survival time in the CRC cohort of patients. Follow-up was defined as the time between diagnosis of PM and death, or the last time of medical appointment. Survival up to 3 years after diagnosis and median survival were estimated (with 95% CI) with each variable included in the present study using the Kaplan–Meier estimator. In all patients who died, death was caused by the CRC, so overall survival (OS) is equivalent, in this series, to cancer-related survival.

Next, the present study focused on the association between the KRAS mutation status and patient survival. The distribution of patient and tumour characteristics between groups of KRAS mutations was compared using the x-squared test. Finally, the effect of the KRAS mutation on survival adjusted for these characteristics was evaluated using Cox proportional hazard regression. *P* < 0.05 was considered to indicate statistically significant difference.

## 3. Results

### 3.1. Patients and Characteristics

A total of 149 patients were included in the study, 85 (57%) men and 64 (43%) women. The mean age was 63 ± 10 years (range: 32–80). The clinicopathological characteristics are shown in Table 1. Of these patients, 46 (30.9%) were treated with 5-fluorouracil schemes and 103 (69.1%) were given bevacizumab or anti-EGFR agents according to the KRAS mutation status. CRS/HIPEC was performed in 36 patients (24%). Five patients (3.3%) were not operated on due to disseminated disease and absence of symptoms of the primary tumour. Surgical treatment was performed on 144 patients: resection of the primary tumour in 130 (87.2%) and palliative nonresection procedure in 14 (9.5%).

The primary tumour was located in the rectum in 21 (14%) patients, 64 (43%) in the left colon, and 64 (43%) in the right colon. MT-KRAS was present in 75 patients (50.3%). In 34 (22.8%) cases, the ECOG Index was 0. The PCI score was between 1 and 10 in 46 patients (30.9%), between 11 and 20 in 63 (42.3%), and was higher than 20 in 40 (28.8%). Mucinous histopathological type was diagnosed in 44 (29.5%) tumours. In 77 patients (51.7%), PM were synchronous with the primary tumour. In 61 patients (40.9%), PM were the sole focus of metastatic disease.

### 3.2. Long-Term Survival

**Figure 1:**
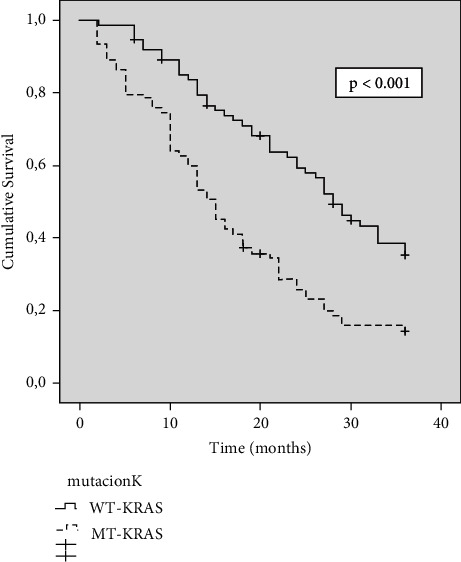
Kaplan–Meier survival function curve in the entire cohort according to KRAS mutation status. The horizontal bar denotes median survival.

Kaplan–Meier estimations of OS at 36 and 60 months after diagnosis were 24% and 17%, respectively (median: 21 months; 95% CI: 16–25). The results of the univariate survival analysis are shown in Table 1. Likelihood of survival at 36 months was higher in patients with WT-KRAS tumours (35% vs. 14%; *P* < 0.001) (Figure 1), ECOG 0 patients (47% vs. 17%; *P* < 0.001), N0 patients (51% vs. 18%; *P* < 0.001), patients with tumours of classic adenocarcinoma histology (31% vs. 9%; *P* < 0.001). Patients with PCI 1–10 showed a likelihood of survival at 36 months (56%), which was longer than that found in patients with PCI 11–20 (16%) and that found in patients with PCI >20 (nil) (*P* < 0.001). Also, the likelihood of survival was higher in patients who underwent HIPEC (64% vs. 11%; *P* < 0.001) and in patients who were treated with 5-FU + bevacizumab/cetuximab chemotherapy programs (52% vs. 9%; *P* < 0.001).

In the univariate analysis (Table 1), the risk of dying was significantly higher in patients with ECOG 1-2 Index (hazard ratio (HR), 2.49), N1-2 tumours (HR, 2.84), tumours of mucinous histologic type (HR, 2.54), low grade of differentiation (HR, 2.36), MT-KRAS tumours (HR, 2.18), and high PCI (HR, 7.43). The risk was significantly lower in patients with metachronous PM (HR, 0.65).

### 3.3. Variation of Survival Results Is Observed Depending on the KRAS Status

**Figure 2:**
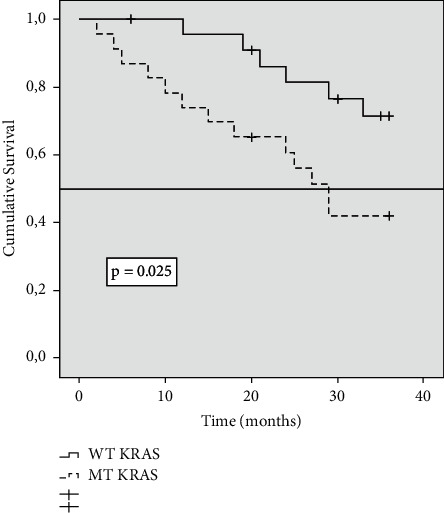
Kaplan–Meier survival function curve in patients with PCI 1–10 according to KRAS mutation status. The horizontal bar denotes median survival.

**Figure 3:**
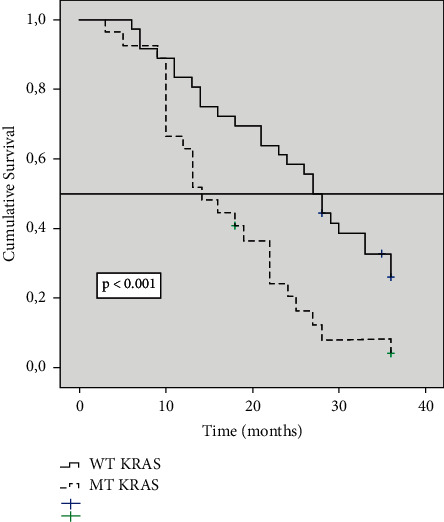
Kaplan–Meier survival function curve in patients with PCI 11–20 according to KRAS mutation status. The horizontal bar denotes median survival.

**Figure 4:**
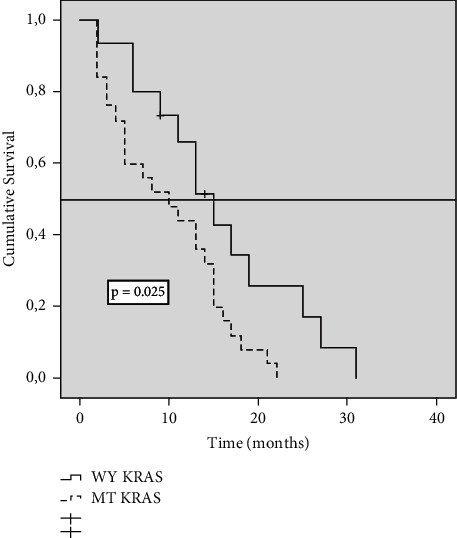
Kaplan–Meier survival function curve in patients with PCI >20 according to KRAS mutation status. The horizontal bar denotes median survival.

We observed that in the three categories in which PCI was classified, the likelihood of survival was different according to the KRAS gene status. In the PCI 1–10 group, survival at 36 months was 71% in patients with WT-KRAS tumours versus 41% in patients with MT-KRAS tumours (*P*=0.025) (Figure 2). In patients with PCI 11–20, survival was 26% in patients with WT-KRAS tumours versus 4% in patients with MT-KRAS tumours (*P* < 0.001) (Figure 3). In patients with PCI >20, survival at 36 months was nil, but at 28 months, survival in the WT-KRAS group was 8% versus 0 in MT-KRAS (*P*=0.025) (Figure 4). In the group of 36 patients treated with CRS/HIPEC, we found no difference in survival at 36 months between WT-KRAS and MT-KRAS patients (*P*=0.91).

Mutation frequency in KRAS was analysed with regard to clinical and histopathological factors (Table 2). The presence of the MT-KRAS was more frequent in N positive tumours (54.1%) (*P*=0.04), tumours with poor grade of differentiation (65.8%) (*P*=0.02), and mucinous tumours (73.8%) (*p* < 0.001).

In the multiple regression analysis (Table 3), the factors that showed an independent prognostic value were KRAS mutation, *N*+ tumours, tumour grade of differentiation, ECOG 1-2 Index, and PCI.

## 4. Discussion

Survival of patients with CRC and PM remains poor despite new chemotherapy and treatment schemes. A meta-analysis of 14 randomized trials involving 10,553 patients with metastatic CRC, 1374 (13%) of whom had peritoneal metastases, reported that patients with metastases only in the peritoneum had shorter survival rate than those with a single nonperitoneal site when treated with systemic treatment (median OS 16.3 months vs. 20 months, respectively) (*P* < 0.0001). For patients with more than one site of metastases, survival was worse if the peritoneum was included, with a median OS of 12.6 months, compared to 15.7 months when the peritoneum was not included (*P* < 0001) [7].

Until now, patient's prognosis has been assessed using clinicopathological factors, such as age, clinical symptoms, extra peritoneal metastases, and histology, including differentiation grade and state of the lymph nodes. These factors and the PCI [12] have been combined into clinical scores to estimate the expected survival, the risk of peritoneal relapse, and planning for the most adequate treatment [9, 10, 13]. However, appropriate selection of patients remains unclear, resulting in certain patient relapse with consequent minimal survival.

The present study verified that PCI showed the most determinant prognostic value. This factor provides a direct measure of tumour burden in the peritoneum, and it is logical that it is the main indicator of the expected survival of these patients. Any factor to be taken into account as a prognostic parameter in patients with PM should provide complementary information to that provided by PCI.

In our study, KRAS mutation exhibited high prognostic importance and independent value. Patients with MT-KRAS tumours had a 2.144 times higher risk of dying than patients with WT-KRAS tumours, and the likelihood of survival at 36 months was higher (35% vs. 14%) (*P* < 0.001). Furthermore, the survival within the subclasses of the PCI varied depending on the KRAS status.

Tumours of mucinous histological type showed significantly lower survival than classic adenocarcinoma. The association between mucinous histological type and poor outcome has been recognized in other studies. This type of tumour shows a particular tendency to peritoneal dissemination and resistance to current drugs [14]. In our study, we have grouped tumours with signet ring histopathology and other mucinous tumours together due to the small sample size analysed, although it is known that the outcome of signet ring cell is worse [15]. A high ECOG score was associated with poorer survival. Most of the authors consider that current practice for patient selection should include it, as it provides an estimate of clinical status.

Tumours located in the right colon had lower OS than those in the left colon or rectum. However, location did not maintain prognostic value in the multivariate analysis. Primary tumours localized in the right colon have been associated with lower survival, particularly in patients with stage IV disease [16]. It is thought that the worst progression of proximal tumours could be attributed in part to the frequency with which genetic alterations, such as BRAF and KRAS mutations, microsatellite instability, and CpG island methylator phenotype, are detected [17]. In addition, tumours with mucinous histology occur more frequently here than at other sites.

Poor grade of differentiation was associated with low OS and high prognostic value. This association has been well recognized previously [9, 13]. In addition, we found a close relationship between tumour grade and KRAS mutation. The incidence of KRAS mutation was higher in poorly differentiated tumours (33.4% vs. 17.6%) (*P*=0.02). These two factors demonstrated independent prognostic weight in the multivariate analysis, which indicates that the prognostic information provided by both factors is complementary.

PM diagnosed at follow-up had higher OS than those detected synchronously with the primary tumour, although the difference had no influence on the multivariate analysis. Other publications have reported higher OS for PM synchronous with the primary tumour [18]. It is possible that the discrepancy is due to the type of patients studied. Our study was performed on a general series of patients with PM. Studies attributing increased survival to synchronous PM have been performed in patients who are candidates for CRS/HIPEC.

The significance of KRAS mutational status is complex. The presence of mutated KRAS gene has been associated with resistance to EGFR-targeted agents. Patients with mutated KRAS gene should not be treated with anti-EGFR therapy. But, patients with wild-type KRAS tumours can be treated with bevacizumab or anti-EGFR therapy. It is not known how this fact can affect the survival. However, in previous publications, the worst survival of patients with MT-KRAS tumours has been found to be independent of the chemotherapy treatment and it has been attributed to more aggressive tumour behaviour [11, 19]. Our data corroborate the prognostic implications associated with mutation of KRAS and its informative value as the prognostic factor in CRC patients.

Few data are known on the impact that the presence of KRAS gene mutations may have in patients with CRC and PM. In an initial publication, Gillern [20] studied 23 patients with peritoneal carcinomatosis and found no difference in survival between patients with MT-KRAS and WT-KRAS tumours. Massalou [21] and Mo [22] analysed KRAS mutations in patients treated with CRS/HIPEC. There was no statistically significant relationship between KRAS status and the median survival. In contrast, four other more recent multicentre studies have found shorter survival in MT-KRAS tumours in which CRS/HIPEC was performed [18, 23–25]. Morgan et al. [23] analysed the value of KRAS mutation in 47 patients undergoing CRS/HIPEC. KRAS mutation was associated with decreased relapse-free survival, but there was no difference in OS. They concluded that KRAS mutation is an independent marker of early recurrence in patients undergoing CRS/HIPEC for CRC and may identify patients who do not derive benefit from this high-risk procedure. Arjona-Sanchez [18] analysed the risk factors for survival in a series of 77 patients treated with CRS/HIPEC and found that KRAS mutational status was an independent factor for OS (HR: 2.024; *P*=0.045).

The success of the therapeutic approach depends on an optimal selection of patients. Most published studies on prognostic factors in patients with PM of colorectal cancer have sought to develop scoring index for the selection of patients who are candidates for CRS/HIPEC. All of these indexes are based on clinical and pathological features. None of these indexes shows an exact correlation with the subsequent evolution of patients. Current scores do not consider any molecular characteristics of the treated tumour. Nowadays, the therapeutic strategy planning for malignant diseases often includes a molecular profile, going towards a personalized treatment [18]. The data from our study confirm the prognostic value of KRAS determination in patients with PM from CRC. KRAS mutation status provides information that may enhance risk stratification.

The present study is limited by the small number of patients, the retrospective design, and being performed in a single hospital. The main criticism in terms of the conclusion of the overall prognostic value of KRAS mutation would be the uneven distribution of high-risk features between the WT-KRAS and MT-KRAS groups: PCI score, multiple site metastases, grade of differentiation, which tend to favour the WT-KRAS group. This is an inherent problem of such study design, and the multivariate analysis partly addressed this issue. Despite these limitations, the mutational status of the KRAS gene has demonstrated a strong association with survival. The data obtained support the inclusion of KRAS mutational status determination in the multifactorial prognostic index in future prospective studies.

## 5. Conclusion

The mutational status of the KRAS gene has a strong association with survival and prognostic utility in patients with CRC and peritoneal metastases.

## Figures and Tables

**Table 1:** Patient and tumour characteristics and survival estimate rates (95% CI) at 36 months after diagnosis.

	Patients (%) (total = 149)	Cumulative survival 36 months (%)	Median	*P* value	HR	95% CI
*Sex*						
Men	85 (57%)	24	22	0.72	1	
Women	64 (43%)	24	20		1.07	0.73–1.56

*Age (years)*						
<50	17 (11.4%)	41	27	0.14	1	
50–69	80 (53.7%)	26	23		1.76	0.88–3.52
>70	52 (34.9%)	16	16		1.29	0.66–2.54

*Location*						
Right colon	64 (43%)	15	17	0.21	1	
Left colon	64 (43%)	32	25		0.86	0.49–1.51
Rectum	21 (14%)	19	24		0.69	0.46–1.05

*ECOG Index*						
0	34 (22.8%)	47	36	<0.001	1	1.47–4.19
1-2	115 (77.2%)	17	17		2.49	

*T stage*						
T3	68 (45.6%)	26	24	0.42	1	
T4	81 (54.6%)	23	19		1.16	0.79–1.7

*N stage*						
N0	27 (18%)	51	NR	<0.001	1	1.55–5.19
N1-2	122 (81%)	18	21		2.84	

*Time of diagnostics*						
Synchronic	77 (51.7%)	14	18	0.03	1	
Metachronic	72 (48.3%)	26	26		0.65	0.44–0.96

*Site of metastases*						
Peritoneum only	61 (40.9%)	33	25	0.01	1	
Peritoneum + liver	51 (34.2%)	27	21		2.08	1.26–3.41
Peritoneum + lung	8 (5.4%)	12	15		2	0.89–4.5
Multiple	29 (19.5%)	6	13		1.27	0.8–2.01

*Grade of differentiation*						
Well to moderate	111 (74.5%)	30	25	<0.001	1	
Poor	38 (25.5%)	6	12		2.36	1.56–3.57

*Histologic type*						
Classical adenocarcinoma	105 (70.5%)	31	27	<0.001	1	
Mucinous	44 (29.5%)	9	12		2.54	1.7–3.8

*KRAS status*						
WT-KRAS	74 (49.7%)	35	28	<0.001	1	1.48–3.2
MT-KRAS	75 (50.3%)	14	15		2.18	

*Peritoneal cancer index*						
1–10	46 (30.9%)	56	NR	<0.001	1	
11–20 (a)	63 (42.3%)	10	22		7.43	4.16–13.24
>20 (b)	40 (28.8%)	0	13		2.59	1.53–4.36

*Chemotherapy treatment*						
5-FU-based programs plus bevacizumab/cetuximab	103 (69.1%)	52	NR	<0.001	1	
5-FU-based programs	46 (30.9%)	9	14		2.86	1.92–4.24

*Primary tumour resection*						
Yes	130 (87.2%)	27	22	<0.001	1	
No	19 (12.8%)	0	15		2.29	1.34–3.90

*HIPEC*						
Yes	36 (75.8%)	64	60	<0.001	1	
No	113 (24.2%)	11	15		5.45	2.98–10.04

The log-rank test was used to calculate *P* values. HR: hazard ratio; 95% CI: 95% confidence interval; NR: not reached. Hazard ratio compares PCI: (a) PCI 11–20 versus PCI 1–10; (b) PCI >20 versus PCI 11–20.

**Table 2:** KRAS mutation status according to patient and tumour characteristics.

	WT-KRAS (*n* = 74)	MT-KRAS (*n* = 75)	*P* value
*Sex*			
Men (*n* = 85)	43 (50.6%)	42 (49.4%)	0.46
Women (*n* = 64)	31 (48.4%)	33 (51.6%)	

*Age*			
<50 (*n* = 17)	9 (53%)	8 (47%)	0.62
50–69 (*n* = 80)	42 (52.5%)	38 (47.5%)	
>69 (*n* = 52)	23 (44.2%)	29 (55.8%)	

*Location*			
Right colon (*n* = 64)	28 (43.7%)	36 (56.3%)	0.32
Left colon (*n* = 64)	33 (51.5%)	31 (48.5%)	
Rectum (*n* = 21)	13 (61.9%)	8 (38.1%)	

*ECOG Index*			
0 (*n* = 34)	20 (58.8%)	14 (41.2%)	0.15
1-2 (*n* = 115)	54 (46.9%)	61 (53.1%)	

*T stage*			
T3 (*n* = 68)	33 (48.5%)	35 (51.5%)	0.46
T4 (*n* = 81)	41 (50.6%)	40 (49.4%)	

*N stage*			
N0 (*n* = 27)	18 (66.6%)	9 (33.4%)	0.04
N1-2 (*n* = 122)	56 (45.9%)	66 (54.1%)	

*Time of diagnostics*			
Synchronic (*n* = 77)	36 (46.7%)	41 (53.3%)	0.28
Metachronic (*n* = 72)	38 (52.8%)	34 (47.2%)	

*Site of metastases*			
Peritoneum only (*n* = 61)	36 (59%)	25 (41%)	0.08
Peritoneum + liver (*n* = 51)	26 (51%)	25 (49%)	
Peritoneum + lung (*n* = 8)	3 (37.5%)	5 (62.5%)	
Multiple (*n* = 29)	9 (31%)	20 (69%)	

*Grade of differentiation*			
Well to moderate (*n* = 111)	61 (55%)	50 (45%)	0.02
Poor (*n* = 38)	13 (34.2%)	25 (65.8%)	

*Histologic type*			
Classic adenocarcinoma (*n* = 105)	61 (58%)	43 (42%)	<0.001
Mucinous (*n* = 44)	12 ((27.7%)	32 (73.8%)	

*Peritoneal cancer index*			
1–10 (*n* = 46)	23 (50%)	23 (50%)	0.15
11–20 (*n* = 63)	36 (57.1%)	27 (42.9%)	
>20 (*n* = 40)	15 (37.5%)	25 (62.5%)	

*Primary tumour resection*			
Yes (*n* = 130)	69 (53%)	61 (47%)	0.02
No (*n* = 19)	5 (26.3%)	14 (73.7%)	

*Chemotherapy treatment*			
5-FU-based programs plus bevacizumab/cetuximab (*n* = 103)	57 (55%)	46 (45%)	0.51
5-FU-based programs (*n* = 46)	17 (37%)	29 (63%)	

*HIPEC*			
No (*n* = 113)	50 (44.2%)	63 (55.8%)	0.01
Yes (*n* = 36)	24 (66.6%)	12 (33.4%)	

A *χ*2 test was used to calculate the *P* values.

**Table 3:** Predictive factors of survival by Cox multivariate analysis.

	*P* value	HR	95% CI	
			Inferior	Superior
MT-KRAS	0.001	2.144	1.342	3.424
N positive	0.033	2.382	1.071	5.296
ECOG 1-2	0.006	2.469	1.289	4.732
PCI	0.000			
PCI 11–20 vs. PCI 1–10	0.000	6.218	3.172	12.188
PCI >20 vs. PCI 11–20	0.004	2.409	1.319	4.400
Poor grade differentiation	0.003	2.426	1.354	4.347
Mucinous type	0.106	1.476	0.902	2.368

HR: hazard ratio; 95% CI: 95% confidence interval.

## Data Availability

The datasets used and/or analysed during the current study are available from the corresponding author upon reasonable request.
